# Effectiveness and Safety of Transdermal Buprenorphine for Acute Postoperative Pain Management Following Mandibular Resection: A Prospective Observational Study

**DOI:** 10.7759/cureus.79634

**Published:** 2025-02-25

**Authors:** Ayesha Ahmad, Khizar Ali Khan, Emaan Hamid, Muhammad Fahad Nisar, Aneesa Rehmat

**Affiliations:** 1 Pathology, Khyber Medical University, Peshawar, PAK; 2 Medicine and Surgery, Rehman Medical College, Khyber Medical University, Peshawar, PAK; 3 Medicine and Surgery, Isra University, Islamabad, Islamabad, PAK; 4 Medicine and Surgery, Pak International Medical College, Khyber Medical University, Peshawar, PAK; 5 Medicine and Surgery, Muhammad College of Medicine, Khyber Medical University, Peshawar, PAK

**Keywords:** analgesia, mandibular resection, opioid alternatives, patient satisfaction, postoperative pain, transdermal buprenorphine

## Abstract

Background

Managing postoperative pain after mandibular excision and reconstruction remains challenging, and using conventional opioid medications has a risk of side effects.

Objective

This study aimed to evaluate the effectiveness and safety of the transdermal buprenorphine patch in managing postoperative acute pain following mandibular resection and reconstruction in a real-world clinical setting.

Methodology

A prospective, observational study was conducted, involving 108 patients who underwent mandibular resection or reconstruction between January and December 2023. Following surgery, participants received transdermal buprenorphine patches, and the visual analog scale (VAS) was used to measure pain severity at baseline and 6, 12, 24, and 48 hours after application. Additionally, documented were adverse effects, the usage of rescue analgesia, and patient satisfaction. The data was examined using paired t-tests.

Results

The average VAS pain score before surgery was 7.83 ± 1.27, however, it dropped dramatically to 5.24 ± 1.35 after six hours, 4.18 ± 1.28 at 12 hours, 3.07 ± 1.10 at 24 hours, and 2.23 ± 1.05 at 48 hours. Nausea (9.26%) and dizziness (5.56%) were the most frequent side effects, with 74.07% of patients reporting no negative symptoms. 70.37% of patients did not need any rescue analgesics, and 85.19% of patients expressed satisfaction or high satisfaction with the way their pain was managed. Significant pain reductions were seen at all time periods using paired t-tests (p < 0.001).

Conclusion

The transdermal buprenorphine patch is a safe and effective alternative for managing acute postoperative pain following mandibular surgery.

## Introduction

Postoperative treatment relies heavily on effective pain control, especially for complicated surgeries like mandibular reconstruction and excision [1]. Owing to considerable soft tissue and bone manipulation, these procedures are usually accompanied by severe acute pain, which might impede healing owing to physical and psychological stress [2]. Systemic opioids are often used in traditional postoperative pain management techniques. Although they are effective, these drugs have the potential to cause side effects including respiratory depression, nausea, and reliance [3]. Maximizing analgesia and minimizing side effects has led to the investigation of alternative modalities [4].

Transdermal drug delivery methods have attracted attention among these substitutes because of their capacity to administer drugs in a regulated, prolonged manner [5]. For the treatment of moderate to severe pain, the transdermal buprenorphine patch has shown promise [6]. Because of its strong analgesic effects and high lipid solubility, buprenorphine, a partial μ-opioid receptor agonist and κ-opioid receptor antagonist, is well suited for transdermal administration [7]. Compared to conventional systemic opioids, it has a number of benefits, such as a lesser chance of respiratory depression, a ceiling effect for opioid-induced adverse effects, and a lower potential for misuse [8].

Although buprenorphine patches have been shown to be effective in treating chronic pain, nothing is known about how well they work for immediate postoperative pain [9]. Because mandibular resection and reconstruction surgeries need a pain management approach that promotes early mobility and recovery of mouth functions, in addition to requiring considerable nociceptive input, they provide a distinct clinical situation [10,11]. There is a lot of clinical interest in the transdermal buprenorphine patch's possible function in this situation [2].

Buprenorphine exhibits a favorable pharmacological profile; however, its effectiveness in managing acute postoperative pain following major oral and maxillofacial procedures remains inadequately characterized, warranting further investigation. This study aimed to evaluate the clinical effectiveness and safety of transdermal buprenorphine for postoperative pain control after mandibular resection and reconstruction, utilizing pain intensity scores, adverse event incidence, and patient-reported satisfaction as primary outcome measures.

## Materials and methods

Study design and setting

This prospective, observational study was conducted at the Pakistan Institute of Medical Science (PIMS) Islamabad, Rehman Medical Institute (RMI) Peshawar, and Khyber Teaching Hospital (KTH) Peshawar. The study spanned a duration of one year, from January 2023 to December 2023.

Inclusion and exclusion criteria

Inclusion criteria for this study were patients aged 18 years or older undergoing mandibular resection and reconstruction surgery, who were experiencing moderate to severe postoperative pain requiring opioid analgesia, and who were willing to provide written informed consent and comply with the study protocol. Exclusion criteria included patients with known hypersensitivity to buprenorphine or its components, severe hepatic or renal impairment, pregnancy or lactation, history of substance abuse or opioid dependency, and conditions or treatments interfering with pain assessment (e.g., severe cognitive impairment).

Sample size

The study employed convenience sampling to recruit participants, with a total of 108 patients included based on their availability and fulfillment of the inclusion criteria during the study period. To minimize selection bias, standardized eligibility criteria were applied uniformly across all study sites. Furthermore, regression analysis was performed to adjust for potential confounders influencing pain perception and opioid response.

Intervention

Transdermal buprenorphine patches were administered following FDA-approved protocols for persistent moderate to severe pain. Typically, these patches release buprenorphine at a rate of 20 μg/hour for seven days in chronic pain management. However, for this study, the dose was adjusted to 10-20 μg/hour based on patient-specific factors such as body weight and pain intensity to address acute postoperative pain. The patches were placed on clean, dry, non-irritated, and hairless skin (e.g., upper arm or back), avoiding injured or scarred areas. Each patch remained in place for 48 hours, ensuring consistent analgesia during the critical initial postoperative period.

Data collection

Pain severity was measured using the visual analog scale (VAS) at baseline (before patch application) and at 6, 12, 24, and 48 hours post-application. Adverse effects such as nausea, dizziness, constipation, and skin irritation at the application site were documented throughout the 48-hour period. The requirement for rescue analgesia, whether opioid or non-opioid, was recorded to evaluate the patch’s effectiveness. Patient satisfaction with pain management was assessed at the end of the 48-hour period.

Statistical analysis

Descriptive and inferential statistical analyses were conducted. Continuous variables were expressed as mean ± standard deviation (SD), while categorical data were presented as frequencies and percentages. The normality of data distribution was assessed using the Shapiro-Wilk test. Pain score changes over time were analyzed using paired t-tests for within-group comparisons. Between-group differences were assessed using independent t-tests. Regression analysis was performed to adjust for potential confounders such as age, BMI, comorbidities, and baseline pain scores. A p-value of <0.05 was considered statistically significant. All statistical analyses were conducted using SPSS software (version 27, IBM Corp., Armonk, NY), with visualizations created in MS Excel (version 2016, Microsoft Corp., Redmond, WA).

Ethical approval

The study protocol was reviewed and approved by the Ethical Review Board of Khyber Medical University, Peshawar (IRB approval number: DIR/KMU-EB/DMS/22-38). To minimize bias in data collection, all study personnel were trained on standardized pain assessment and patient reporting methods. Written informed consent was obtained from all participants before inclusion, ensuring confidentiality and adherence to ethical research standards.

## Results

Demographic and clinical characteristics

The study included 108 patients with a mean age of 45.61 ± 12.58 years, comprising 62 males (57.41%) and 46 females (42.59%). Among the surgeries, 52 (48.15%) received mandibular reconstruction and 56 (51.85%) had mandibular resections alone. On the visual analog scale (VAS), the average preoperative pain level was 7.83 ± 1.27; the average surgical length was 4.31 ± 1.24 hours (Table 1).

**Table 1:** Demographic and clinical characteristics of study participants

Characteristics		Values
Age in years	Mean ± SD	45.61 ± 12.58
Gender; n(%)	Male	62 (57.41)
	Female	46 (42.59)
Procedure type; n(%)	Mandibular resection alone	56 (51.85)
	Mandibular reconstruction	52 (48.15)
Mean duration of surgery (hours)	Mean ± SD	4.31 ± 1.24
Preoperative pain score (VAS)	Mean ± SD	7.83 ± 1.27

Pain intensity and reduction

Measuring pain intensity with VAS ratings, the transdermal buprenorphine patch greatly lowered the value with time (Table 2). At baseline, the mean pain score was 7.83 ± 1.27; it dropped to 5.24 ± 1.35 at 6 hours, 4.18 ± 1.28 at 12 hours, 3.07 ± 1.10 at 24 hours, and 2.23 ± 1.05 at 48 hours. This shows a continuous and significant pain decrease over the postoperative period.

**Table 2:** Pain intensity (VAS scores) at different time intervals

Time Interval	Mean Pain Score (± SD)	Range (min-max)
Baseline (pre-application)	7.83 ± 1.27	6.50-9.00
6 hours post-application	5.24 ± 1.35	3.10-7.45
12 hours post-application	4.18 ± 1.28	2.55-6.85
24 hours post-application	3.07 ± 1.10	1.55-5.48
48 hours post-application	2.23 ± 1.05	1.05-4.02

Adverse effects

**Figure 1:**
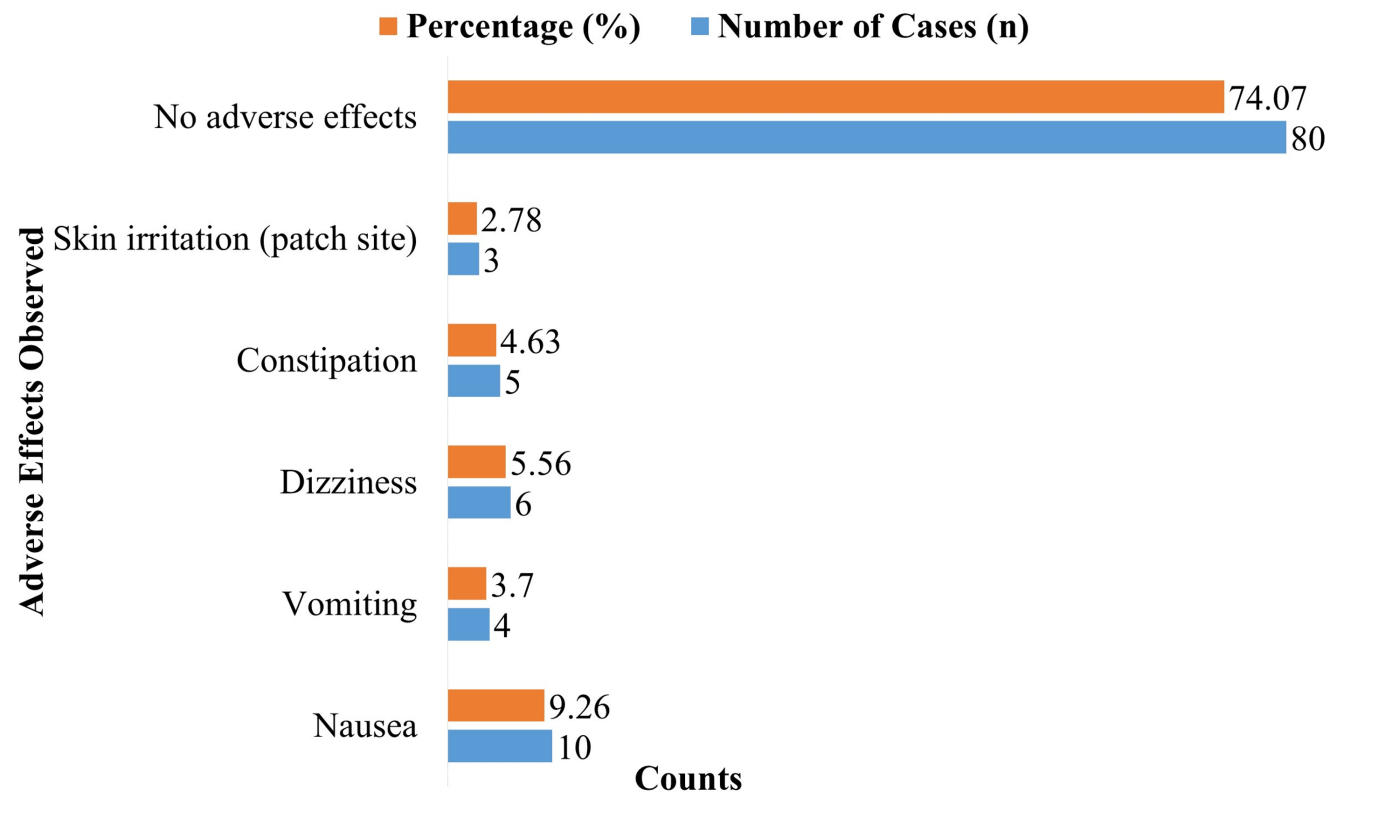
Adverse effects observed during the study period

A total of 80 participants (74.07%) had no negative side effects throughout the research (Figure 1). Nausea was the most frequently reported adverse effect (9.26%), followed by dizziness (5.56%), constipation (4.63%), vomiting (3.70%), and skin irritation at the patch site (2.78%). This demonstrates the transdermal buprenorphine patch's general safety and tolerability.

Patient satisfaction

**Figure 2:**
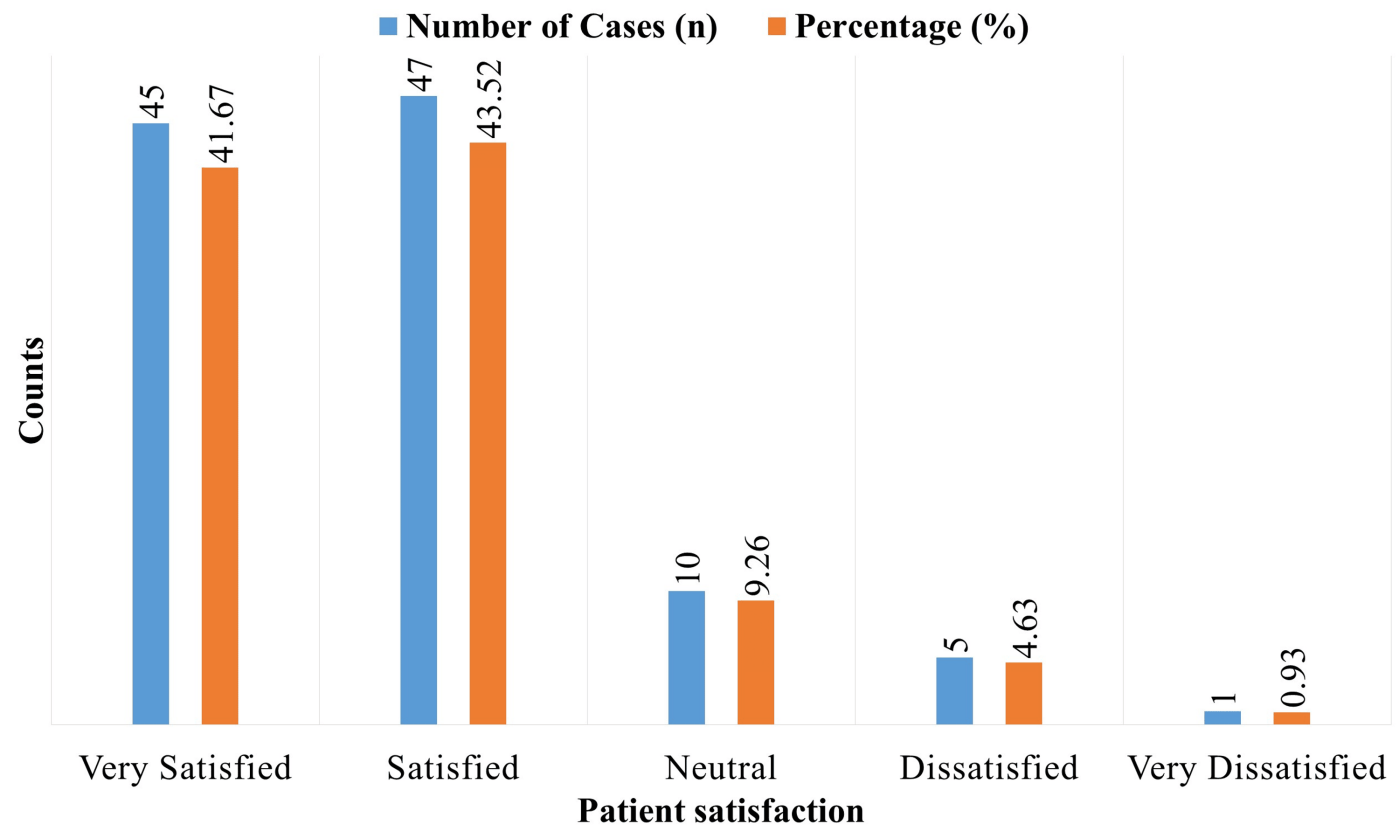
Patient satisfaction with pain management (post-48 hours)

A total of 45 patients (41.67%) reported being extremely happy with their pain management 48 hours after application, whereas 47 patients (43.52%) reported being satisfied (Figure 2). Overall, patient satisfaction with pain treatment was good. A lower percentage reported feeling neutral (n=10; 9.26%), unhappy (n=5; 4.63%), or extremely dissatisfied (n=1; 0.93%), suggesting that the transdermal buprenorphine patch was generally well-received for its ability to control pain.

Use of rescue analgesia

**Figure 3:**
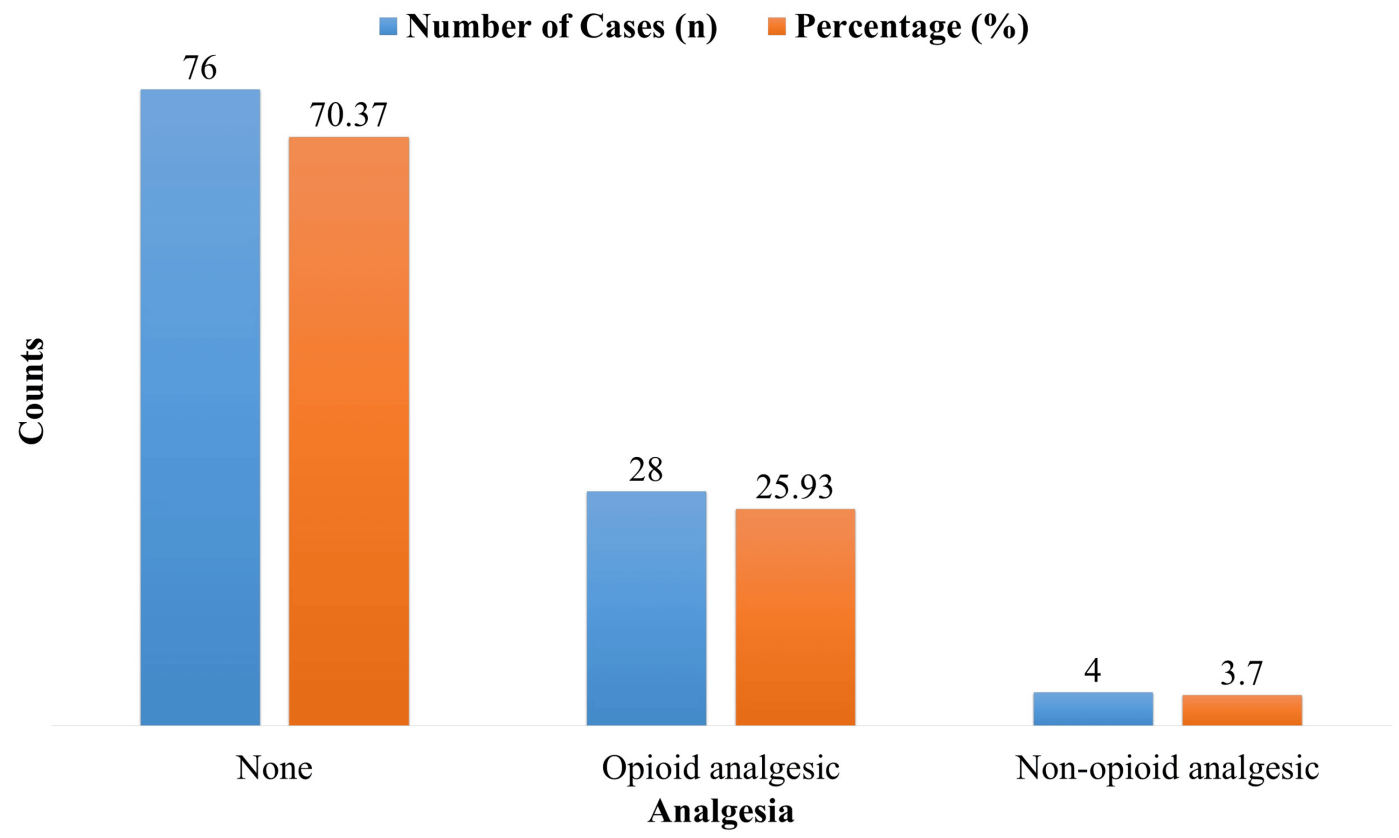
Use of rescue analgesia (opioid or non-opioid) in study participants

Four patients (3.70%) used non-opioid analgesics, while 28 patients (25.93%) required opioid analgesia for additional pain relief. The average opioid consumption among these patients was 12.5 ± 3.8 mg of morphine equivalent over 48 hours. The majority of patients (70.37%) did not require additional analgesia, indicating effective pain control with transdermal buprenorphine (Figure 3).

Statistical analysis of rain reduction

Following the transdermal buprenorphine patch administration, the paired t-test findings show a substantial decrease in pain at all-time intervals (Table 3). The average pain score difference between baseline and 6 hours was 2.59 ± 1.15 (p < 0.001), and after 12 hours (3.65 ± 1.12, p < 0.001), 24 hours (4.76 ± 1.05, p < 0.001), and 48 hours (5.60 ± 1.02, p < 0.001), there were additional decreases. Significant pain reductions were also seen in comparisons between the following time periods; all p-values were less than 0.001, suggesting that the patch continued to be effective for the whole 48 hours.

**Table 3:** Paired t-test results for pain score reduction at different time intervals

Comparison Interval	Mean difference ± SD	t-value	p-value
Baseline vs. 6 hours post-application	2.59 ± 1.15	18.72	<0.001
Baseline vs. 12 hours post-application	3.65 ± 1.12	24.05	<0.001
Baseline vs. 24 hours post-application	4.76 ± 1.05	32.19	<0.001
Baseline vs. 48 hours post-application	5.60 ± 1.02	38.77	<0.001
6 hours vs. 12 hours post-application	1.06 ± 0.85	12.43	<0.001
12 hours vs. 24 hours post-application	1.11 ± 0.76	14.68	<0.001
24 hours vs. 48 hours post-application	0.84 ± 0.68	11.73	<0.001

Multiple linear regression analysis (Table 4) demonstrated that the baseline pain score was the strongest predictor of postoperative pain at 48 hours (β = 0.72, p < 0.001), indicating that patients with higher initial pain experienced greater reductions. The duration of surgery also showed a minor but significant effect on pain reduction (β = 0.15, p = 0.035), suggesting that longer surgical procedures might be associated with slightly higher postoperative pain levels. However, gender (β = 0.05, p = 0.578) and age (β = -0.02, p = 0.063) were not significant predictors of pain reduction. The model explained 64% of the variance (R² = 0.64, p < 0.001), confirming that baseline pain and surgical duration play a key role in postoperative pain outcomes.

**Table 4:** Multiple linear regression analysis for pain score reduction (dependent variable: pain score at 48 hours) P-values <0.05 were significant.

Predictor variable	Beta coefficient (β)	Standard error (SE)	95% Confidence interval (lower bound, upper bound)	t-value	p-value
Baseline pain score	0.72	0.08	0.56, 0.88	9.00	<0.001
Duration of surgery (hours)	0.15	0.07	0.01, 0.29	2.14	0.035
Gender (male)	0.05	0.09	-0.12, 0.22	0.56	0.578
Age (years)	-0.02	0.01	-0.04, 0.00	-1.88	0.063

## Discussion

Our research on the effectiveness and safety of the transdermal buprenorphine patch for treating acute postoperative pain after mandibular excision and reconstruction reveals a significant decrease in pain severity. The initial pain score was 7.83 ± 1.27, and 48 hours after application, it had progressively dropped to 2.23 ± 1.05. This illustrates how well the patch works to provide long-lasting pain relief. These results are in line with earlier research that showed buprenorphine, a partial opioid agonist, provides longer-lasting pain relief with fewer adverse effects than conventional opioids [12]. Its usage in acute postoperative situations is further supported by research by Jawanda et al. (2021) who found a comparable decrease in pain ratings when transdermal buprenorphine was used for chronic pain control [13].

Around 74.07% of patients in our research reported no side effects, indicating a very low prevalence of adverse effects. Among the most frequent side effects, nausea was reported by 9.26% of patients, followed by constipation (4.63%) and dizziness (5.56%). These numbers are in line with prior transdermal buprenorphine research, which has typically shown a low frequency of serious side effects [2,14]. The idea that transdermal buprenorphine offers a safer substitute for oral or intravenous opioids, which are often linked to increased rates of respiratory depression, reliance, and overdose, is supported by the low frequency of major side effects.

With 85.19% of patients expressing that they were either extremely pleased or satisfied with their pain treatment, patient satisfaction in our research was also high. This outcome is in line with recent research that found that most patients having significant jaw operations were satisfied with buprenorphine's analgesic effects, praising its simplicity of use and efficacy in comparison to conventional opioids [15]. With 70.37% of patients requiring no rescue analgesia, our study's excellent satisfaction rate demonstrates the transdermal buprenorphine patch's capacity to successfully control pain while reducing the need for additional analgesics.

With a mean difference of 5.60 ± 1.02 after 48 hours, the statistical analysis of pain reduction, as shown by the results of the paired t-test, showed significant decreases at every time point. These findings are in line with other research that looked at transdermal buprenorphine for postoperative pain management in oral and maxillofacial surgeries. In those studies, comparable pain reductions were seen after major jaw procedures like reconstruction and mandibular resection [16,17]. To sum up, our study's findings confirm the increasing amount of data indicating that the transdermal buprenorphine patch is a safe and efficient substitute for postoperative pain treatment.

Study strengths and limitations

This study's strengths include its prospective design, validated pain measurement technique (VAS), and comprehensive statistical analysis, ensuring reliable data on the effectiveness and safety of transdermal buprenorphine for postoperative pain management. The inclusion of a diverse patient sample (n = 108) enhances the generalizability of findings to similar patient populations undergoing mandibular resection and reconstruction. To minimize bias, we employed standardized pain assessment tools, strict inclusion criteria, and predefined protocols for data collection and patient monitoring. Additionally, independent reviewers conducted data analysis to enhance objectivity.

As an observational study, this research assesses the real-world effectiveness of transdermal buprenorphine rather than its efficacy, which would require a randomized controlled trial (RCT) with a placebo or active comparator. The study’s design does not allow for causal inferences. Our focus on pain management within 48 hours postoperatively means that long-term analgesic effectiveness and patient outcomes were not assessed. The absence of a control group and the use of convenience sampling introduce potential biases, which may limit the generalizability of findings.

Given these limitations, a future randomized clinical trial incorporating placebo or active comparators, randomization, and blinding would provide a more rigorous assessment of transdermal buprenorphine’s efficacy. Further research with larger sample sizes and extended follow-up periods is essential to confirm these findings and explore long-term safety, patient satisfaction, and opioid-related adverse effects.

## Conclusions

Transdermal buprenorphine patches have been shown to significantly reduce pain ratings at many time periods (6, 12, 24, and 48 hours) after mandibular resection and reconstruction, suggesting that they are an effective way to manage postoperative acute pain. Despite the fact that nausea was the most frequent side effect, most patients expressed high levels of satisfaction with the way their pain was managed. The effectiveness of the patch as a main pain management technique was further shown by the significant percentage of patients who did not need supplemental rescue analgesics. Although further research is required to validate its long-term effectiveness and safety, these findings imply that transdermal buprenorphine may be a potential substitute for conventional opioid-based analgesia in this clinical context for controlling postoperative pain.
